# Finding hens in a haystack: Consistency of movement patterns within and across individual laying hens maintained in large groups

**DOI:** 10.1038/s41598-018-29962-x

**Published:** 2018-08-17

**Authors:** C. Rufener, J. Berezowski, F. Maximiano Sousa, Y. Abreu, L. Asher, M. J. Toscano

**Affiliations:** 1 ZTHZ, Division of Animal Welfare, University of Bern, Bern, Switzerland; 2 Veterinary Public Health Institute, University of Bern, Bern, Switzerland; 3 Centre for Behaviour and Evolution, IoN, Newcastle University, Durham, England; 4 National Centre for Animal and Plant Health, San José de las Lajas, Cuba

## Abstract

We sought to objectively quantify and compare the recorded movement and location patterns of laying hens within a commercial system. Using a custom tracking system, we monitored the location within five zones of a commercial aviary for 13 hens within a flock of 225 animals for a contiguous period of 11 days. Most hens manifested a hen-specific pattern that was (visually) highly consistent across days, though, within that consistency, manifested stark differences between hens. Three different methods were used to classify individual daily datasets into groups based on their similarity: (i) Linear Discriminant Analysis based on six summary variables (transitions into each zone) and total transitions; (ii) Hierarchical Clustering, a naïve clustering analysis technique, applied to summary variables and iii) Hierarchical Clustering applied to dissimilarity matrices produced by Dynamic Time Warping. The three methods correctly classified more than 85% of the hen days and provided a unique means to assess behaviour of a system indicating a considerable degree of complexity and structure. We believe the current effort is the first to document these location and movement patterns within a large, complex commercial system with a large potential to influence the assessment of animal welfare, health, and productivity.

## Introduction

Animals of various species have structured behavioural routines with variations relating to individuality. Poultry have been found to show differences in environmental preferences^1–4^ which are likely driven by traits intrinsic to the individual such as boldness (also known as proactivity/reactivity)^5^, fearfulness^6^, and stress reactivity^7,8^. In humans, consistent individual differences exist in movement patterns^9^ with comparable patterns for diverse animal species^10^. However, the extent to which there are consistent individual differences of movement patterns for chickens is unknown, a knowledge gap which represents an important, fundamental research need within poultry species.

The existence of individual movement and location patterns of poultry likely has implications for applied systems as well. Modern commercial poultry systems (particularly in Europe and North America) are increasingly moving towards large, open systems with flocks of 500 to over 100,000 animals within a single barn where each animal could theoretically access every area without limitation. Limited work examining hens’ use of outside areas suggests the existence of sub-populations within the flock^1–3,11^ that likely translates to differential effects on the health and welfare of the individual^12,13^. However, to the authors’ knowledge, no work exists examining individual animals within the interior of large, non-cage barns. It is also known that in relatively small groups (e.g. 20 hens), hens will form a well-defined and stable social hierarchy which is used to determine access to key resources^14–16^. As group size increases, birds seem to adopt a different social system characterized by decreased acts of aggression^17^. How this changing social structure relates to altered movements (e.g. to avoid specific individuals or hoarding and/or use of high value resources) in large commercial settings is unknown.

Given the pattern of larger group and total farm size within the poultry sector and agricultural system as a whole, producers must increasingly rely on technological innovation to monitor important variables such as environmental conditions, equipment operations, and characteristics of the animals themselves. The use of technology for these purposes is collectively known as Precision Livestock Farming^18^ and has the potential to bring considerable benefits to the producers, consumer, and the animals themselves^19^. One particular use of Precision Livestock Farming is surveillance of individual animals using metrics for physiological variables (e.g., body temperature) as well as movement and location. However, these developments have largely excluded poultry production for several reasons that were previously summarized^20^. In brief, reasons include logistical issues such as the relatively small mass and size of poultry (compared to cattle or swine) which limit the size of equipment that can be placed on the animal. The characteristics of housing for poultry also complicates individual monitoring as it may involve multiple vertical layers (or even stacked tiers) necessitating tracking in three dimensions.

In summary, the modern commercial laying industry has substantial potential to deliver improvements to hen welfare, health, and productivity with deployment of appropriate technology. Given the complex social structures and motivations underlying laying hen behaviour, laying houses also offer a rich setting for the study of complex social structures and influencing factors. With this focus in mind, our research group developed a custom-designed tracking system that allowed the registration of individual hens within five key areas or zones of a commercial, multi-tier aviary system. Preliminary analysis of collected data unexpectedly revealed large differences in patterns of movements between hens as well as consistent patterns within hens (i.e., over multiple, consecutive days). The current effort, which was exploratory in nature, investigated methodological tools that could objectively quantify the recorded movement and location patterns to allow comparison across and within hens. We hypothesized that daily movement and location patterns would be more similar for an individual bird than between different birds, i.e., an individual hen’s movement and location pattern from one day would be more similar to her own pattern on another day compared to a pattern of a different hen.

## Methods

All experimental animal work was approved by the Bern Kantonal authority (BE-31/15) and all methodologies were performed in accordance with the relevant national and kantonal guidelines and regulations. Observations were conducted within a commercial laying hen house previously described^21^. In brief, the barn contains a three-tiered aviary system (Bolegg Terrace, Krieger AG, Ruswil, Switzerland) located in the middle of the animal area and then divided into 20 identical pens (450 cm × 700 cm × 230 cm). Each pen was separated with a horizontal wire mesh panel (openings 1 cm × 2 cm) that allowed visual, olfactory, and auditory contact between adjacent pens. The pen floor was covered with an approximate 5 cm depth of wood shavings (resupplied above old bedding approximately every two weeks). Each pen had access to a separate (outdoor) wintergarden (9.32 m^2^) that was fully enclosed with a solid roof and wire mesh sides. The floor of the wintergarden was covered with wood shavings and sand. Pens were equipped with round metal perches (14 perches per pen; length: 230 cm; 14 cm / hen; outer diameter: 3.2 cm), nipple drinkers, and an automatic chain feeding system providing standard laying hen feed *ad libitum*. Fresh feed was delivered every two hours during lighted hours. Artificial light was provided from 02:00:00 to 17:00:00 with a transitional period of 5 and 20 minutes in duration beginning at 02:00:00 and 16:40:00, respectively. Natural daylight was provided through windows on both sides of the barn that operated on an automated curtain system.

The work reported here was part of a larger study that required a combination of two genetic lines (Lohmann Selected Leghorn (LSL), Lohmann Brown (LB)) to be co-housed within each pen in a specific format. The density of animals in relation to overall space, feeders, drinkers, and other pen features were within the range required for commercial Swiss laying hen systems. The pen used in the current study contained 15 LSL and 210 LB hens of which 15 hens (4 LB, 11 LSL) were selected for observation using the tracking system over either a 7- or 11-day period beginning at 61 weeks of age. Two hen recordings were not used due to equipment malfunctioning, leaving 13 hen recordings for final analysis.

**Figure 1:**
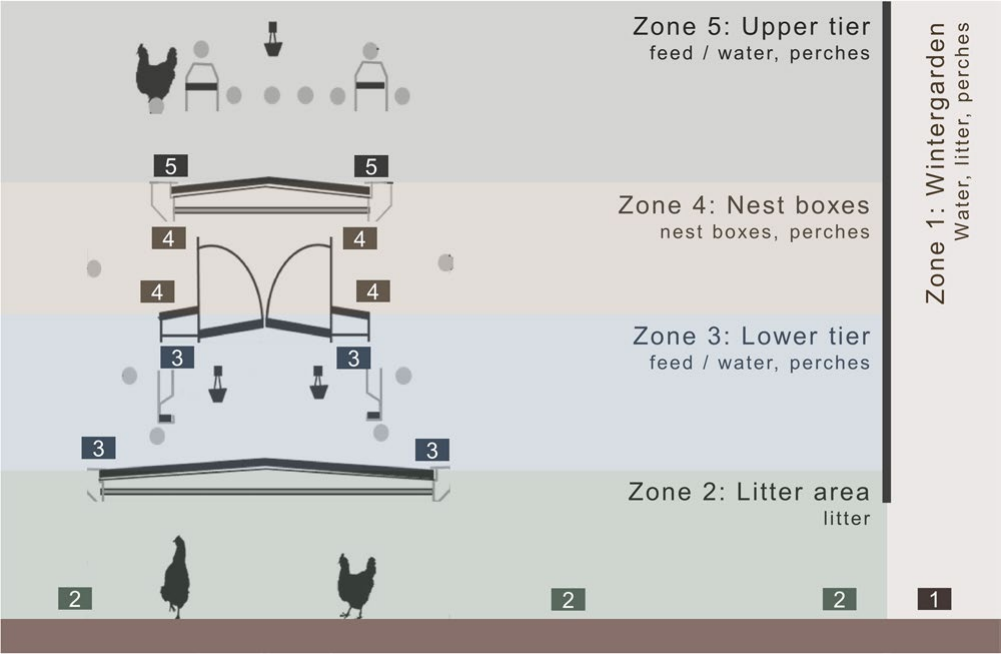
Representation of the aviary system and the five coverage zones showing available resources in each zone as well as emitter positioning (boxes).

To monitor animal location and movement, we developed a custom-designed system that employed emitter units generating infrared beams with embedded codes specific to one of five, pre-defined zones of the aviary (Fig. 1)^22^. Emitters were placed at specified locations within each zone at specific heights chosen to maximize spread of the infrared beams by reducing signal blockage. Focal hens wore a receiving unit (widest diameter: 2.2 cm) mounted within a plastic container attached to a commercial legband (mass: legband with container: 6.3 g; receiver: 2.1 g; battery 1.3 g) that recorded the coded infrared beam along with a time-date stamp at a frequency of 1 Hz. At the conclusion of the observation session, focal hens were collected, receivers removed and data downloaded as a CSV file. From the registration of each zone with the time-date stamp, a dataset containing the time at which hens transitioned between zones and the amount of time spent in each zone was created for each bird in the study. It is important to note that the system did not monitor any behaviour other than transitioning between zones, including activity within a particular area. Hence, use of the phrase ‘hen activity’, ‘movements’, or ‘transitions’ refers to movement between zones unless otherwise specified.

To validate the system, four LB hens were observed directly by two human observers on either side of the pen for two, 2-hour sessions per hen (i.e., eight, 2-hour sessions with two birds observed per session). Comparison of data recorded by the system and that of the direct observers found the zone was correctly assigned 94% of the time. Furthermore, the time-date stamp of changing zones recorded by the tracking system differed by 8+/− 25.6 s (mean +/− SD) in comparison to the direct observations.

All analyses were performed within R^23^ using packages and functions as described. Since studies of individual interior movements of chickens had not been undertaken previously, and the extent of individual differences between hens unknown, data visualization was considered to be an important first step in the analysis. Plots of overall movement patterns from sensor output were generated to provide an overview of individual hen movement and location patterns over the observation period. Beyond the charts provided here, we developed an array of visualization tools including: graphs, tables and other representations of collected data. The tools have been developed in an R-Shiny application (https://shiny.rstudio.com/application) and are accessed through a web portal that is available for viewers to visualize the complete dataset (i.e., all 13 hens across the entire study period) by contacting the corresponding author. Initial observation of the generated graphs (including those not presented here but available via the R-shiny tool) was used as the basis for determining relevant variables to quantify and compare individuals, as well as the types of analysis appropriate for data of this nature.

To test the hypothesis that daily movement patterns were more similar within hens than between hens, we created daily data sets (one for each complete day of observation for each bird). Three different methods were used to classify the individual daily datasets into groups based on their similarity: (i) Linear Discriminant Analysis (LDA) based on summary variables (described below); (ii) Hierarchical Clustering (HC), a naïve clustering analysis technique, applied to summary variables and iii) Hierarchical Clustering applied to dissimilarity matrices produced by Dynamic Time Warping (DTW). To allow for all hens to be used for the classification procedure, only seven contiguous days (the first seven days in the associated plots, i.e. August 23^rd^, 2017 thru August 29^th^, 2017) were used as four hens had incomplete datasets (i.e., missing days) due to procedures of the larger study. Using data from the 13 tracked animals with seven complete days of observation, 91 individual daily datasets were created and used for classification. Each dataset was labelled with the bird identification number and day of the time series, e.g., dataset 7.3 contained data for bird 7 on day 3.

Frequency of visits to each of the five zones and total transitions/day were selected as six key variables for classification using LDA or HC from 02:00:00 to 16:59:59. Linear Discriminant Analysis^24,25^ was used to evaluate the effectiveness with which these variables could characterise different individuals. Linear Discriminant Analysis identifies clusters within one or more dependent variables in relation to a categorical, independent variable which has different classes. In this case, the independent variable was bird ID where each bird ID was a class with multiple observations (i.e.7 hen days) on the same hen. The mean and distribution values of data across dependent variables were used to form combinations which maximized the separation of data points from different classes of the independent variable (i.e., hen days from birds with different IDs) while minimising separation of data points within the same class (i.e., hen days from birds with the same ID). Based on LDA, the probability of each data point (i.e., in our case, hen day) belonging to each class of the independent variable was calculated and the maximum probability used to assign a posterior (predicted) class. The LDA procedure was implemented using the MASS package^25^ in R (version 0.98.507). The six summary variables were initially included as dependent variables, and then sequentially dropped and re-entered into the LDA if the model was improved by 5% or more of variance by including that explanatory variable. Predictions of the hen’s identity from the data for each day were made using the final LDA model. The number of times (and percent) that the LDA correctly classified daily data sets for the correct bird ID was calculated.

Using the same summary variables as with LDA, HC^26^ was used to test the similarity of daily data sets of individual hens. Data on these six variables were passed to a hierarchical clustering algorithm implemented in R function hclust using the Ward D method. Hierarchical Clustering is a method which examines the similarity of values of data points where the Ward D method uses a bottom-up approach. Starting from each single data point, the near nearest data point is found and a decision made to combine these into a cluster or not in a stepwise manner. The decision for pairing clusters at each step is based on minimising the error from the squares. The algorithm is complete when all data points are combined into a single cluster.

The second technique used for cluster analysis was DTW^27^, package dtwclust^28^. Dynamic Time Warping serves to cluster complete time series based on dissimilarities between the structure of individual time series. Each daily data set was converted to a time series of the zone location for the bird for each second during the day. Each daily time series had 54,000 data points covering the same period used for the LDA and HC procedures (i.e., 02:00:00 to 16:59:59). The pairwise dissimilarity between all pairs of time series was then calculated by DTW. The method generated a 91 × 91 dissimilarity matrix containing distance metrics for each pairwise comparison. The window size for the DTW algorithm was set at approximately 10% (5000 L). A full description of the DTW method can be found at: https://github.com/asardaes/dtwclust . The Ward D method of HC was applied to the dissimilarity matrix of the DTW.

For both clustering analyses (HC and DTW), generated dendrograms were cut into n to n + 2 groups to explore whether groupings identified naively would match individual identifications. Based on the best groupings (most data points from the same individual placed in the same cluster) from each clustering analysis, the number of times each individual was placed in the same grouping was calculated.

## Results

**Figure 2:**
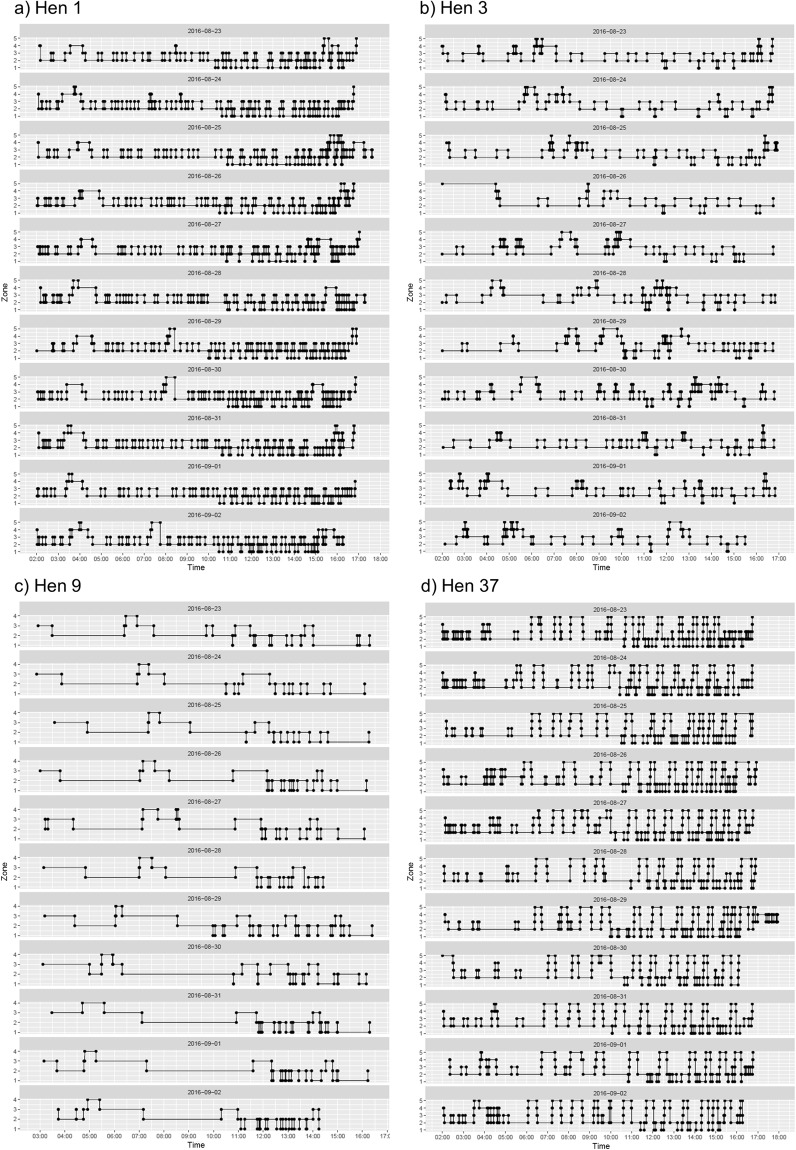
Location graphs of four focal hens over 11 consecutive days. Each panel represents a single hen (1, 3, 9, 37). Within each graph, each line (top to bottom) represents location data for a single day where lights were on between approximately 0200 and 1700. The Y-axis represents the zone in which the animal was present and the X-axis represents the time of day.

**Figure 3:**
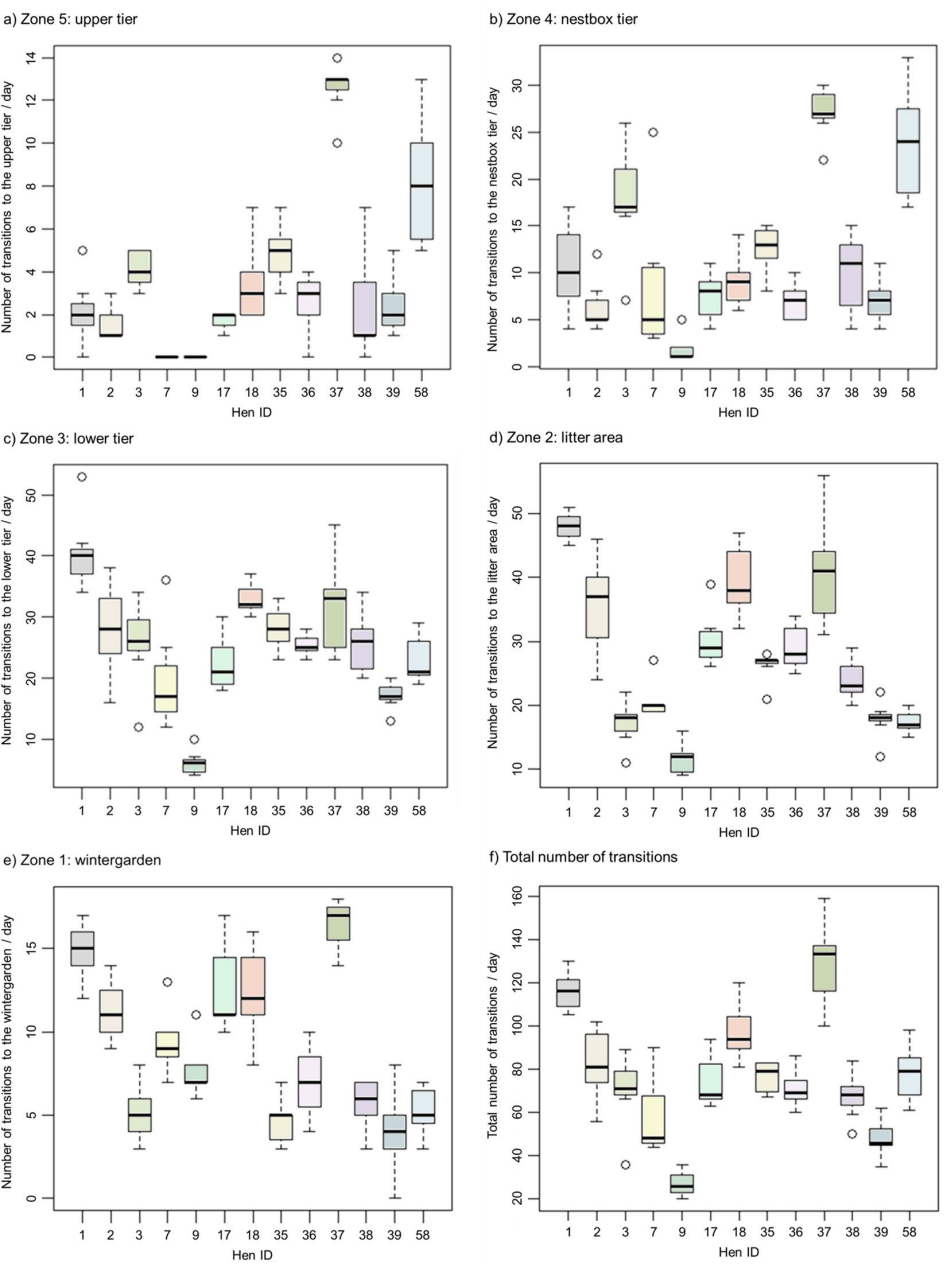
(**a**–**f**) Box and whisker plot of all hens over the entire observation period for frequency of visits. The areas are: (**a**) Zone 5 (upper tier), (**b**) Zone 4 (nestbox tier), (**c**) Zone 3 (lower tier), (**d**) Zone 2 (litter area), (**e**) Zone 1 (wintergarden), and (**f**) the total number of transitions.

Visualization of collected data demonstrated remarkable consistency within individuals as well as starkly different profiles between hens. Movement patterns for four hens over the same consecutive 11 days are provided for comparison (Fig. 2). The four individuals were selected from observed hens that were included in the differentiation effort as they represented relative extremes of the observed patterns. Most hens manifested a hen-specific pattern that was highly consistent (visually) across days, though manifested stark differences between hens. Some visual features that stand out between hens when examining the movement plots are: patterns of specific, repeating transitions at a relatively similar rate/time; extended duration of time within a single specific zone; lack of entry into an area; and isolated periods of activity within extended periods of inactivity. These and other features are most striking when occurring repeatedly across days at similar times of the day. For instance, hen 9 did not enter the upper tier (zone 5) though it spent extended periods of time in the litter area (zone 2) and visited the nestbox tier (zone 4) at approximately the same time and for the same duration each day. Based on data from all hens (independently of whether they were used for differentiation), the mean and standard error, as well as the proportion of the total transitions for the selected six key variables (transitions to each zone and total transitions) were calculated (Table 1). Most transitions occurred into the litter area (zone 2) (36%) with the least occurring into the upper tier (zone 5) (5%). Box plots for daily transitions of individual hens are provided for each variable (Fig. 3).

**Table 1:** Average of total daily visits to each zone, and the average total daily visits for all zones (included are the mean, standard error (SE) and percent of total).

Zone	Mean	SE	% of total
1: Wintergarden	8.97	0.47	11.71%
2: Litter area	27.52	1.19	35.94%
3: Lower tier	25.09	0.99	32.77%
4: Nestbox tier	11.46	0.84	14.97%
5: Upper tier	3.54	0.38	4.62%
Total transitions	76.57	2.97	—

**Figure 4:**
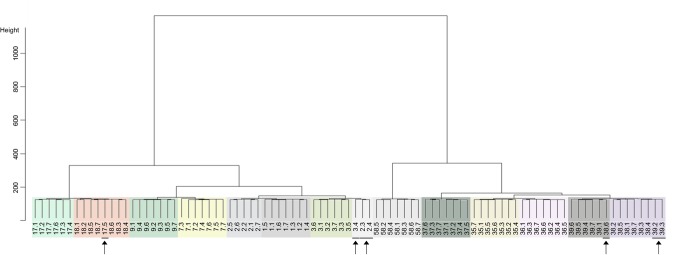
Dendogram of classification resulting from Hierarchial Clustering of selected variables. The identifying numbers represent the Hen ID and the associated date. Clusters of grouped animals are surrounded by a colored box (i.e., across all days) to ease comparison. We have indicated animal.days that are incorrectly grouped.

**Figure 5:**
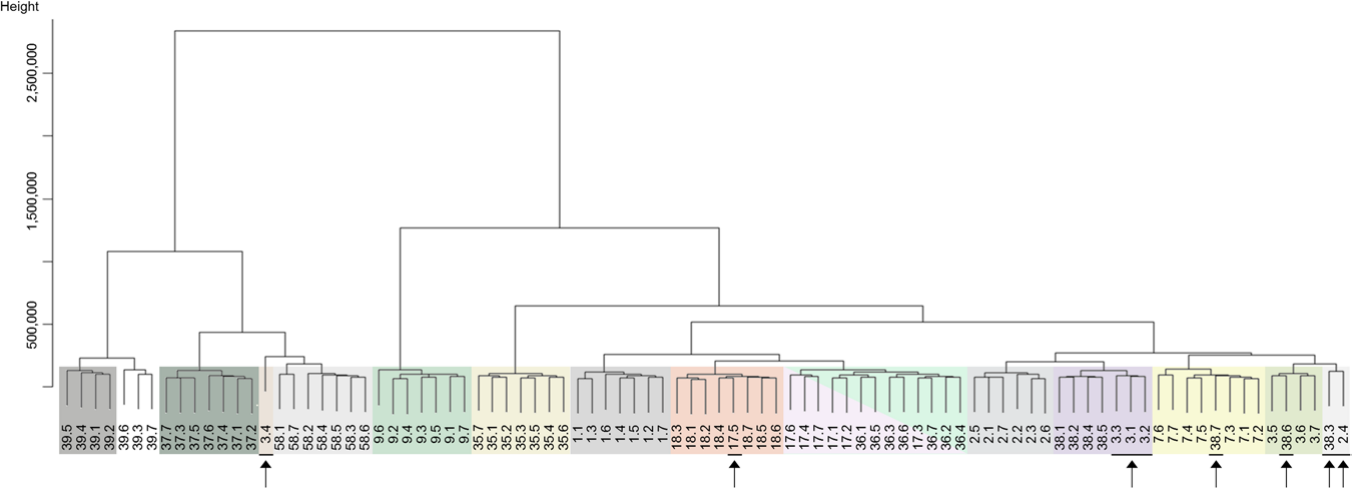
Dendogram of classification resulting from Dynamic Time Warping. The identifying numbers represent the Hen ID and the associated date. Clusters of grouped animals are surrounded by a colored box (i.e., across all days) to ease comparison. We have indicated animal.days that are incorrectly grouped.

Linear Discriminant Analysis correctly classified 84.6% of the daily data sets including 5 of the 13 hens in which all daily datasets were correctly classified (Table 2). The final model included transitions to the litter area (zones 2), lower tier (zone 3), upper tier (zone 5), and total transitions. Using the same summary variables for HC, 92.3% of the daily datasets where correctly classified including eight of the 13 hens in which all daily datasets were correctly classified (Table 2). The final model used all six variables. Dynamic Time Warping correctly classified 90.1% of the daily datasets including nine of the 13 hens in which all daily datasets were correctly classified (Table 2). Resulting dendograms are provided for HC (Fig. 4) and DTW (Fig. 5).

**Table 2:** The number and proportion of days individual hens were correctly grouped.

Hen ID	Classification Method^i^					
	Linear Discriminant Analysis		Hierarchical Clustering		Dynamic Time Warping	
	number	%	number	%	number	%
1	7	100%	7	100%	7	100%
2	4	57%	5	71%	6	86%
3	6	86%	6	86%	3	43%
7	6	86%	7	100%	7	100%
9	7	100%	7	100%	7	100%
17	7	100%	6	86%	6*	86%
18	5	71%	7	100%	7	100%
35	6	86%	7	100%	7	100%
36	4	57%	7	100%	7*	100%
37	7	100%	7	100%	7	100%
38	5	71%	6	86%	4	57%
39	7	100%	5	71%	7^†^	100%
58	6	86%	7	100%	7	100%
Overall	77	84.60%	84	92.3%	82	90.1%

^i^A total of seven days were used for the procedure. ^*^Hens 17 and 36 were grouped together. ^†^39 was placed in a group which further divided into two subgroups.

## Discussion

Our study investigated movement and location patterns of individual hens within a large, commercial group of laying hens. Based on published literature investigating hen behaviour and social structures within relatively small groups of hens (e.g., less than 20 hens), we predicted a degree of consistency within hens. In excess of our expectations, the range of consistency for movement and location patterns across and within hens was more extreme than anticipated. In response to our initial observations of the patterns via generated visual plots of movement and location, we sought to quantify the observed consistency in terms of intra- (within hen, i.e., across days of the same hen) and inter-hen (between hens) variation and use the generated metrics to differentiate and compare individual hens.

The number of movements to specific zones and in total provided the most visually consistent pattern and thus was a primary area of our focus. For instance, hens 1 and 37 were the two most active transitioning animals with the highest mean total movements of all hens. Despite similarly high transition rates, hen 1 rarely visited the upper tier (zone 5) whereas hen 37 transitioned frequently to this zone, ranging from 0 to 4 and 10 to 14 times per day, respectively. We also observed consistent hourly patterns across days. As an example, hen 1’s presence in the upper tier (zone 5) was almost exclusively at the beginning or end of the lighted hours (i.e., absent midday), whereas hen 37 transitioned continually throughout the day, with the greatest concentration after approximately 0600 hours. The contrasts between these two specific hens represent how two, similarly active animals can manifest very different patterns of activity.

Beyond the upper tier, visits to other specific zones also offered differentiating features. For example, both hens 1 and 37 had similarly high transition rates into the wintergarden (zone 1). Hen 9, which had the lowest number of total transitions across all animals and never entered the upper tier (zone 5), made a relatively moderate number of transitions to the wintergarden (zone 1). Although our principal focus here was transitions, the tracking system allowed for a range of additional measures to be quantified (e.g., duration in individual zones, time first appearing in a zone, etc.), which can be accessed via the R Shiny tool. For instance, hen 9 also spent a relatively large duration of the day in the wintergarden (zone 1) compared to hens 1 and 37, (i.e., total duration in the wintergarden over the 11 day period – Hen 1: 84,059 sec; Hen 9: 132,241 sec; Hen 37: 66,117 sec), typically towards the end of the day just before the pophole to the wintergarden was closed.

Given the clear visual differences between hen location patterns, a principal aim was to develop objective methods to differentiate and compare individual hens. The three utilized methods had a relatively high success rate (>=85%) though results were dependent on the hen and method of differentiation. For instance, hens 1 and 37, both with relatively high transition rates and consistent visual patterns across days, were classified correctly for all observed days. Hen 9, despite a comparatively lower overall rate of transitions, had a relatively consistent visual pattern where transition rates were relatively infrequent in the first half of the day and then increased from approximately 1100 hours (one hour after the popholes opened). The consistency, despite the low activity levels, led to hen 9 being classified correctly 100% of observed days for each method (as with hens 1 and 37). In contrast, the number of transitions for hen 3 was relatively mid-range, though had an inconsistent visual pattern across days, which likely contributed to the poor classification result. Thus, the differentiation tools are capable of providing a useful metric to compare hens in terms of similarity across time and animal, and should also be able to identify periods (e.g., hours, days) when there are changes to hens’ movement pattern.

Data from the four selected hens also illustrate the potential strengths and weaknesses of the differentiation techniques. Hens 1, 9, and 37 had consistent numbers of transitions and similar (hourly) patterns of motion and activity across days that likely contributed to the perfect classification rate. Hen 3 was correctly classified 86% of the time using LDA and HC, though the rate of success fell by 50% for DTW, likely a consequence of the highly variable patterns for this hen. Specifically, day 4 for hen 3 (i.e., 3.4 using the described nomenclature) seemed to be a particular outlying day for this hen with several defining features that contrasted with the other evaluated days. For days 1 through 3, the hen began the day in the nestbox tier (zone 4) and then descended to the litter area (zone 2) within 30 minutes, whereas day 4 began on the upper tier (zone 5) where she remained for approximately three hours. Subsequently, on days 5 through 7, the hen began the day on the litter area (zone 2). The impact of these contrasts on the specific differentiation method is most apparent when examining the associated dendograms. The dendogram for DTW, the method most sensitive to variations in time and location, divided days for hen 3 into three groupings that appear to match the distinctions highlighted above, i.e., the three groups being: days 1 through 3, 4, and 5 through 7. In contrast, HC grouped all days for hen 3 together with the exception of 3.4, which was grouped in the next closest group. Interestingly, while the clustering methods provide different outputs that can be interpreted independently, combining interpretation from the two may serve a synergistic benefit in providing a metric of the patterns. In the example of hen 3, while the location of her activity may have varied over days (as suggested by DTW), her overall activity levels remained consistent (as suggested by HC). It is also important to note that DTW used all collected data, whereas HC used a small number of variables or features extracted from the data, differences which might capture different aspects of hen activity. Dynamic Time Warping might have been expected to capture more subtle differences in the sequence of events, whereas the extracted features might have highlighted more gross differences in movement patterns. The fact that both methods can detect consistency within hen’s movements and differences between individuals is promising for future characterization efforts.

An important feature of the tracking system was that, rather than providing coordinates of the hen’s location, the system functioned by reporting whether the hen was or was not within a specific zone. While the system provided less information than a coordinate-based system (e.g., GPS), the individual zones were chosen as they contain different resources which hens use to perform highly motivated, instinctually driven behaviours that are important for hen welfare and farm productivity. For instance, the nestbox located in zone 4 is designed to attract the hen (e.g. by providing a darkened area) to perform her daily egg laying^29^, an event normally preceded by egg-laying behaviour where the hen may investigate the available nestboxes, sometimes repeatedly^30^. The ability to access the nestbox is generally regarded as a powerful motivational drive^31,32^ resulting in frustration when access is denied^33^. Comparable motivational forces also drive hens to perform dust-bathing^34^ (using substrate provided in the litter area (zone 2)), access the wintergarden (zone 1), and seek an elevated position immediately preceding and during the dusk period^35^. The hen would also need to meet her physiological nutritional and water requirements by accessing feed troughs (zones 5 and 3) and nipple drinkers (zones 5, 3, and 1), respectively. In theory, a highly productive animal could remain its entire life in zones 5 and 4 (i.e., upper and nestbox tiers) where it would have access to feed and water, an elevated roosting position, and a nesting location that is part of the designed apparatus to automatically collect and deliver laid eggs for sale. However, the movement and location patterns suggest a far more complex lifestyle and an understanding of the functional significance of the areas may help to explain pattern variation.

As an example where a zone’s resources may provide an explanation for variation in patterns, a hen’s laying cycle will be on a relatively set interval between ovipositions, which normally occur between 0400 and 1100 hours for the majority of hens. The laying pattern appears to manifest in the activity graphs, though with daily- and hen-specific variation. For example, hens 1 and 9 access zone 4 (containing the nestbox) for an approximate 30-min period between 0500 and 0900 which would be an appropriate time interval to allow for nest inspection and egg laying^36^. The exact times are not consistent and may be influenced by a number of variables, including social factors^37^. Within each pen, four nestboxes were available that in theory could each accommodate a maximum of approximately 18 hens (i.e., an estimated 72 hens of the 225 in the pen could be in the nestboxes at any one time) requiring individuals to adjust their laying schedule. Dominant hens would be more likely to access the nest earlier or with less consideration of current nestbox users, whereas less dominant hens may need to wait until a place is available and/or take a less desirable position within the nestbox (e.g., central rather than against a wall^38^). If this were the case, a less dominant hen might be required to make repeated visits to the nestbox area and/or check both sets of nestboxes requiring a brief visit to the other zones to gain access to both sides of the aviary. Although our tracking system does not indicate when the actual egg was laid (or any behaviour other than moving between two zones), activity plots show hens 3 and 37 made repeated, short visits to the nestbox tier whereas hens 1 and 9 made single visits of a long duration.

The timing of nestbox visits may also be influenced by natural physiological rhythms. For instance, hens 7 and 39 (not shown) appeared to access the nestbox tier on a 25.5 h cycle with the exception of one day, a pause that could allow the hen to reset her cycle, as hens do not lay continuously^39,40^. Other observed patterns include a 24.0 h cycle (hens 35, 36, 2, 18) or the absence of a single visit longer than 15 min (hens 37 and 38). In the latter case where no visit of appropriate duration (>15 min) occurs, hens may be laying their eggs outside the nestbox requiring eggs to be retrieved manually by staff. Such mislaid eggs, often laid in the litter area or on the lower tiers, can be a substantial financial burden for producers due to increased labor costs^41^, particularly for large farms. Thus, although our tracking system does not allow for identification of specific behaviours, the capacity to register entry and exit into functionally specific zones offers a powerful tool to assess indicators of animal welfare and productivity. Future work should be conducted over extended periods of time (e.g., months) to determine the resilience of these movement and location patterns and how they are altered in response to illness, stress, age and other factors of interest.

We believe our data and methods offer a unique way to assess metrics of interest that have become common in other livestock species (e.g., dairy cattle, swine) as part of advances in Precision Livestock Farming. Although it does not seem feasible (or cost-effective) for producers to install individual tracking devices on all hens within their flocks, a percentage of the flock serving as sentinels^20^ could provide important data on flock health and productivity. Assessments should be conducted over longer periods of time to determine how sentinel animals can indicate looming problems or inefficiencies, e.g. decreased activity or identifying characteristics of unproductive hens, the number of sentinel animals required, and benefits of the examined metrics within commercial systems. We also believe that the presented metrics would serve researchers and commercial entities seeking to understand how individual variation relates to traits of interest, most critically within the realm of genetics. Although we do not know the true activity levels of individual hens, it’s reasonable to assume that – given the aviary was 3.5 m tall - hens with repeated transitions up and down (i.e., hens 1, 37) would have a larger energy requirement than those remaining within one level for most of the light period (i.e., hen 9). Future systems could incorporate movement and location tracking systems (and associated analyses) with accelerometer technology to provide a more comprehensive assessment of activity. Housing manufacturers and providers of nutrition would also stand to benefit from an improved understanding of how the hens use the provided environment. For instance, hen 1 and 9, having only rarely or never accessed the upper tier, would be consuming feed from the feeder in the lower tier (zone 3) only. In theory, different diets could be provided to the different tiers at different times of day to better target the nutritional needs of these subpopulations.

## Conclusions

In summary, our methods provide a unique means to assess movement and location patterns that was previously not accessible but indicates the presence of a considerable degree of complexity and structure. While relationship and structure of movement and location patterns within hens and across time have been reported previously, those efforts were largely confined to small groups of animals (e.g., less than 20) and thus have little practical or commercial relevance to group housing. In contrast, we believe the current effort is the first to document these movement and location patterns within a large, complex commercial system and thus has enormous potential to influence the assessment of animal welfare, health, and productivity. In addition to demonstrating a structured pattern of individual activity, we also evaluated several methods of differentiating generated data to allow objective comparison across and within hens. The three selected methods performed well with relatively high levels of agreement, though were dependent on the hen and day of observation. Future work will need to re-evaluate these methods over extended periods of time and in relation to variables of interest, e.g., egg production and hen mortality.
